# Antinociceptive and analgesic effect of continuous intravenous infusion of maropitant, lidocaine and ketamine alone or in combination in cats undergoing ovariohysterectomy

**DOI:** 10.1186/s13028-021-00615-w

**Published:** 2021-11-27

**Authors:** Janaina Maria Xavier Corrêa, Raquel Vieira Niella, Jéssica Natália Silva de Oliveira, Alex Costa Silva Junior, Claire Souza da Costa Marques, Taísa Miranda Pinto, Elisângela Barboza da Silva, Suzane Lilian Beier, Fabiana Lessa Silva, Mário Sérgio Lima de Lavor

**Affiliations:** 1 Department of Agricultural and Environmental Sciences, State University of Santa Cruz (UESC), Campus Soane Nazaré de Andrade, Km 16, Rodovia Jorge Amado, Ilhéus, Bahia CEP- 45662-900 Brazil; 2 School of Veterinary Medicine, Campus Pampulha of Federal University of Minas Gerais, Avenue Antônio Carlos, 6627, mailbox 567, Belo Horizonte, Minas Gerais Brazil

**Keywords:** NK1 receptor antagonist, *N*-methyl-d-aspartate, Neurokinin 1, Pain, Sodium channels

## Abstract

**Background:**

Multimodal analgesia consists of the combination of analgesic drugs at low doses to act in different places along the path of pain. Studies with continuous infusion of analgesic drugs in cats are not common. This study aimed to evaluate the analgesic effect of maropitant, lidocaine and ketamine alone or in combination (intravenous bolus + subsequent continuous intravenous infusion) in the management of acute postoperative pain in cats undergoing ovariohysterectomy. Seventy healthy cats undergoing an ovariohysterectomy received a standard anesthetic protocol consisting of acepromazine and morphine, propofol (anesthesia induction), and isoflurane (anesthesia maintenance). The animals were stratified into seven groups (n = 10 in each group): control (CG), maropitant (MG), lidocaine (LG), ketamine (KG), maropitant + lidocaine (LMG), maropitant + ketamine (KMG), and maropitant + lidocaine + ketamine (LKMG). All drugs were injected first as an intravenous bolus and then by continuous intravenous infusion. During surgery, esophageal temperature, respiratory rate, heart rate, oxygen saturation, expired isoflurane concentration, and partial pressure of carbon dioxide at the end of expiration were evaluated at 7 time points. Postoperative pain was evaluated for 6 h after extubation using the visual analogue scale and the UNESP-Botucatu multidimensional composite pain scale for assessing postoperative pain in cats.

**Results:**

Adverse effects related to maropitant, lidocaine and ketamine infusion were not observed. Pain scores were lower in the MG, KG and LG groups when compared to the CG group using both scales. Although pain scores were also lower in all combination groups than CG, more animals in these groups required rescue analgesia compared to MG. This indicates that the postoperative analgesic effect of all drugs, either alone or in combination, confers analgesia, although the combinations did not promote greater analgesia.

**Conclusions:**

Continuous intravenous infusion of maropitant, lidocaine, and ketamine alone induces postoperative analgesic effect in cats undergoing ovariohysterectomy, but combinations of these drugs did not increase the analgesic effect. No adverse effect was observed with any drug or their combination.

## Background

Surgical procedures elicit harmful stimuli, which, if not prevented, can alter the neuroplasticity of the spinal cord and generate central sensitization. This sensitization process involves several receptors and excitatory neurotransmitters in the dorsal horn of the spinal cord. Thus, novel strategies are needed to prevent central sensitization and improve the management of postoperative acute pain [1].

Multimodal analgesia involves the use of multiple analgesic drugs, usually at low doses, with different mechanisms for managing pain [2–5]. Combinations fentanyl or morphine, lidocaine, and ketamine are commonly used for pain management in dogs during surgical procedures and in the postoperative period [6, 7].

Lidocaine blocks sodium channels, preventing the propagation of action potential, and consequently generates sensory and motor blockade for local and regional anesthesia. It can induce local anesthesia and can be used as an analgesic through continuous intravenous (IV) infusion in dogs and humans both intra- and post-operatively [8, 9]. In cats, continuous IV infusion or IV bolus of lidocaine is not frequently used, mainly because of the risk of intoxication and other adverse effects [10, 11].

Ketamine is a dissociative anesthetic that blocks the *N*-methyl-d-aspartate (NMDA) receptors [12] and is currently used in low doses for pain management in dogs [7, 11]. These sub-anesthetic doses decrease central sensitization and allodynia in dogs and cats [11].

Maropitant is a selective antagonist of the neurokinin 1 receptor (NK1) in the central and peripheral nervous systems. It prevents the activation of the NK1 receptor by inhibiting the binding of substance P to the NK receptor and is recommended for use as an antiemetic in dogs and cats [13–15]. As substance P and its receptor, NK1, play important roles in pain modulation, blockade of NK1 receptors might also provide an antinociceptive effect [16]. This antinociceptive effect has already been demonstrated in domestic cats [17].

Continuous IV infusions of analgesic combinations are not commonly done in domestic cats, mainly because of the risk of intoxication and the prolonged recovery period. In contrast, concerns regarding pain are continuously increasing in the field of veterinary medicine. Consequently, newer techniques for multimodal analgesia are being evaluated in all species. Our hypothesis was that the administration of maropitant, lidocaine and ketamine alone or in combination can induce an antinociceptive effect in cats undergoing ovariohysterectomy (OHE). Changes in cardiorespiratory variables were used to evaluate nociception during surgery and two pain scales (Visual Analog Scale and the UNESP-Botucatu multidimensional composite pain scale) were used to evaluate postoperative pain.

This study aimed to compare the analgesic effect of continuous IV infusion of maropitant, either alone or in combination with lidocaine and/or ketamine, to manage postoperative pain in cats undergoing OHE.

## Methods

The study was conducted after obtaining approval by the Ethical Commission in the Use of Animals (CEUA) of the State University of Santa Cruz—UESC, Bahia, in Brazil (protocol 017/15), and in accordance with the guidelines on care and use of laboratory animals issued by the National Council for Animal Experimentation Control in Brazil.

### Animals and groups

Seventy privately owned female cats aged 2.2 ± 1.3 years (range 6 months–8 years) were included in this study. Health status was verified by clinical examination and laboratory tests renal and hepatic function. Only healthy cats were included. Written consent was obtained from all owners. The animals were housed in individual cages at the UESC Veterinary Hospital 1 day before the OHE. The preoperative fasting period was 8 h for food and 2 h for water.

### Study design

The cats were divided into seven groups (10 in each group) by block randomization. Cats received the same final volume as the control group, according to the weight of each animal:

- Control group (CG): IV bolus 1 mL + continuous IV infusion [5 mL/kg/h] of Ringer’s solution with lactate.
- Maropitant group (MG): IV bolus (1 mg/kg) + maropitant (Maropitant citrate, Cerenia®, Zoetis, Brazil) IV continuous infusion (1.67 µg/kg/min).
- Lidocaine group (LG): IV bolus (1.5 mg/kg) + continuous IV lidocaine (lidocaine hydrochloride 2%, Hipolabor Farmacêutica, Brazil) infusion (50 µg/kg/min)
- Lidocaine and maropitant group (LMG): IV bolus (1.5 mg/kg lidocaine + 1 mg/kg maropitant) + continuous IV infusion of lidocaine and maropitant (50 µg/kg/min and 1.67 µg/kg/min, respectively)
- Ketamine group (KG): IV bolus (1 mg/kg) + continuous IV ketamine (Quetamina® injectable, Vetnil Indústria e Comércio de Produtos Veterinários Ltda, Brazil) infusion (10 µg/kg/min)
- Ketamine and maropitant group (KMG): IV bolus (1 mg/kg ketamine + 1 mg/kg maropitant) + continuous IV infusion of ketamine and maropitant (10 µg/kg/min and 1.67 µg/kg/min, respectively)
- Lidocaine, ketamine, and maropitant group (LKMG): IV bolus (1.5 mg/kg lidocaine + 1 mg/kg ketamine + 1 mg/kg maropitant) + continuous IV infusion of lidocaine, ketamine, and maropitant (50 µg/kg/min, 10 µg/kg/min, and 1.67 µg/kg/min, respectively)

### Procedures

The bolus dose (calculated in mg/kg) was diluted in Ringer’s solution with lactate (final volume, 1 mL) and administered intravenously (manually) over one minute. The injections were administered sequentially for cases in which more than one drug was involved (LMG, KMG, and LKMG). After IV bolus administration of drugs, the animals were anesthetized and continuous IV infusion of one or more drugs was immediately initiated. Notably, maropitant was always infused alone, whereas lidocaine and ketamine were infused together in the same syringe. All drugs used for continuous IV infusions were diluted (Ringer’s solution with lactate) to a final volume of 20 mL. Continuous IV infusions were maintained until the end of surgery with two syringe-infusion pumps (Injectomat Agilia infusion pump, Fresenius Kabi, Bad Homburg, Germany).

All cats were premedicated with intramuscular injections of acepromazine (0.2%; 0.05 mg/kg; Syntec, Brazil) and morphine (0.3 mg/kg; morphine sulfate, 10 mg/mL; Hipolabour + Sanval, Brazil). A 24-gauge catheter (Radiopaque Safelet Catheter; Nipro Medical Corporation Produtos Médicos Ltd., Brazil) was inserted into the cephalic vein 20 min after the pre-anesthetic medications and used for drug administration and fluid therapy using Ringer’s solution with lactate (Fresenius Kabi Brazil Ltda) at a rate of 5 mL/kg/h. Immediately after catheterization, anesthetic induction was performed with propofol administered to effect (5 mg/kg; Propotil; BioChimico Indústria Farmacêutica Ltd., Brazil), and the cat’s trachea was intubated (endotracheal tube number 3.0 or 3.5). Isoflurane (1.5 vol.%) (Isoforine; Cristália Prod. Químicos Farmacêutica Ltda, Brazil) with 100% oxygen (300 mL/kg/min) was administered via a non-rebreathing system (Mapleson breathing systems, Jackson Rees) for maintenance anesthesia. Cephalothin (30 mg/kg) (Ceflen; Agila, Brazil) was administered intravenously as the prophylactic antibiotic therapy before the surgery. The animals were placed in the dorsal recumbency position and antisepsis of the surgical site was performed. Ovariohysterectomy was performed via midline incision caudal to the umbilicus by an experienced surgeon in all animals.

The following variables were evaluated: esophageal temperature (T), respiratory rate (RR), heart rate (HR), oxygen saturation (SpO_2_), expired isoflurane concentration (Etiso) (calibrated automatically daily), and partial pressure of carbon dioxide at the end of expiration (EtCO_2_). All values were obtained with a multi-parameter monitor (Mindray BeneView T8). Systolic blood pressure (SBP) was measured using a vascular ultrasonic Doppler (Vascular Portable Doppler; Medmega DV 610). A blood pressure cuff was placed on the proximal third of the radioulnar region and a Doppler crystal placed over the median artery. If the animals presented physiological changes such as hypotension and hypercapnia, physiological support such as medications (dopamine, ephedrine and dobutamine) and assisted ventilation could be provided.

The following time points were used for evaluation during the surgery: M1, preoperatively; M2, after incision of the linea alba; M3, after right pedicle clamping; M4, after left pedicle clamping; M5, after ligation of the uterus body; M6, suture of the abdominal muscles; and M7, at the end of the surgery. Isoflurane concentration was increased or decreased in accordance with the response of autonomic system (HR, RR and SBP) to the surgical stimulus observed by considering an increase of 20% relative to basal values. Not necessarily all three variables (HR, RR and SBP) increased together. The baseline values were collected from the anesthetized animals before the start of the surgical procedure.

### Pain assessment

Pain was evaluated postoperatively by only one evaluator, who was blinded to the treatment performed. Pain assessment was done 1 h after extubation, and then every hour for 6 h. Two pain assessment scales were used to assess the postoperative pain in cats: the visual analogue scale (VAS) and the UNESP-Botucatu multidimensional composite pain scale for assessing postoperative pain in cats [18].

Whenever the value obtained on the multidimensional scale for postoperative pain assessment was ≥ 10 [18] and/or VAS was ≥ 40 mm [19], analgesic rescue was performed with intramuscular injection of 0.2 mg/kg of morphine (morphine sulfate 10 mg/mL; Hipolabour + Sanval, Brazil). At the end of the evaluation period (6 h), 0.2 mg/kg of meloxicam (0.2% Maxicam; Ourofino, Brazil) was administered to all cats, and 0.2 mg/kg of morphine (intramuscular injection) was administered to the animals that did not receive analgesic rescue.

### Statistical analyses

All data collected were analyzed using Prism for Windows (GraphPad Software. La Jolla, CA, USA). The data were tested for normal distribution using the Shapiro–Wilk test. Normally distributed data (HR, SBP, RR, SpO_2_, EtCO_2_, and T) were subjected to two-way analysis of variance, and their means were compared using Bonferroni test. Non-parametric data (pain assessment scale scores, the rescued animals were not removed from analysis) of the groups were subjected to the Kruskal–Wallis test for between-group comparisons. When these groups were compared with the control group, the Mann Whitney test was used; Fisher’s exact test was used to compare the number of rescue treatments. For all tests, the significance level was set at P < 0.05.

## Results

After administration of the pre-anesthetic medication, no animal exhibited vomiting and/or salivation. The duration of the anesthetic and surgical procedures and the time for extubation were similar between the groups, for anesthesia (35.4 ± 1.2 min), surgery (25.3 ± 0.9 min), and extubation (8.8 ± 1.1 min). The body weight of the animals did not differ significantly between the groups (mean 2.9 ± 0.2 kg).

HR of animals in the CG group was significantly higher at the time of ovarian pedicles ligation when compared with those in the LMG, KMG, and LKMG groups. Animals in the MG group had a significantly higher HR than those in the LMG and KMG groups. Animals in the LG group showed no significant difference in HR when compared with the other groups (Table 1). The RR was significantly higher in the CG, MG, and LG groups than in the KMG, LKMG, and KMG groups (Table 1).

**Table 1:** Variables (mean ± standard error of the mean (SEM)) of the physiological parameters observed during the surgical procedure in the different groups

Variables	Groups	Moments						
		M1	M2	M3	M4	M5	M6	M7
HR (bpm)	CG	150 ± 20	160 ± 23	202 ± 24	195 ± 23	183 ± 22	171 ± 24	169 ± 20
	MG	162 ± 22	155 ± 20	181 ± 20^#^	188 ± 12^#^	177 ± 19	172 ± 21	180 ± 17^#^
	LG	146 ± 23	157 ± 25	176 ± 28	178 ± 30	167 ± 25	165 ± 28	164 ± 27
	KG	161 ± 24	148 ± 17	173 ± 16	177 ± 16	171 ± 18	169 ± 20	165 ± 20
	LMG	136 ± 9	127 ± 7*	161 ± 17*	170 ± 20	158 ± 17	151 ± 13	133 ± 16*^#^
	KMG	139 ± 15	138 ± 21	159 ± 26*^#^	164 ± 18*^#^	156 ± 21*	149 ± 17	133 ± 13*
	LKMG	155 ± 23	153 ± 25	174 ± 16*	178 ± 18	174 ± 12	165 ± 17	154 ± 11
SBP (mmHg)	CG	72.2 ± 14.7	93.8 ± 31.6	127.2 ± 47.5	133.5 ± 39.7	113.0 ± 32.1	107.1 ± 31.9	108.5 ± 17.8
	MG	81.5 ± 8.7	96.1 ± 36.7	114.6 ± 25.0	109 ± 19.1	97.1 ± 28.1	91.6 ± 14.9	94.1 ± 17.2
	LG	55.0 ± 11.9	71.2 ± 13.0	96.5 ± 32.7	92.5 ± 37.0	71.0 ± 19.4	81.75 ± 16.1	80.7 ± 16.2
	KG	71.7 ± 15.6	90.0 ± 32.2	110.7 ± 32.4	108.5 ± 33.4	96.2 ± 42.5	95.7 ± 25.6	104.2 ± 32.6
	LMG	67.0 ± 12.7	87.0 ± 27.5	113.7 ± 52.4	119.2 ± 54.1	101.2 ± 46.3	96.0 ± 41.5	87.5 ± 40.4
	KMG	66.8 ± 16.9	83.3 ± 34.9	109.1 ± 53.4	105.3 ± 43.0	94.8 ± 52.9	91.1 ± 47.4	96.0 ± 46.7
	LKMG	66.0 ± 7.0	85.0 ± 22.7	99.5 ± 19.9	101.0 ± 28.9	90.0 ± 26.4	93.2 ± 24.1	90.7 ± 20.2
RR (mpm)	CG	25 ± 6	27 ± 10	29 ± 10	31 ± 10	29 ± 11	26 ± 10	25 ± 7
	MG	39 ± 10^#^	30 ± 9^#^	32 ± 11^#^	33 ± 10^#^	28 ± 12^#^	29 ± 11^#^	27 ± 13^#^
	LG	30 ± 4•	31 ± 5•	30 ± 5•	28 ± 8	26 ± 8	25 ± 8	27 ± 7
	KG	18 ± 9^#^•	18 ± 7^#^•	19 ± 8^#^•	18 ± 8A	18 ± 9	17 ± 8	20 ± 10
	LMG	24 ± 8	16 ± 5	20 ± 7	18 ± 7A	18 ± 8	16 ± 7	16 ± 6
	KMG	23 ± 8^#^	20 ± 9•	19 ± 10*^#^•	18 ± 7*^#^•	15 ± 7*^#^•	14 ± 9*^#^•	12 ± 6*^#^•
	LKMG	22 ± 10^#^	16 ± 8*^#^•	20 ± 9^#^•	19 ± 8*^#^•	19 ± 12	18 ± 12	18 ± 9
SpO_2_ (%)	CG	99.0 ± 1.6	99.1 ± 1.7	99.7 ± 0.4	99.2 ± 0.7	99.5 ± 0.5	99.6 ± 0.5	99.6 ± 0.5
	MG	96.7 ± 1.6	98.2 ± 1.8	98.6 ± 1.3	98.2 ± 1.2	98.2 ± 1.3	98.2 ± 2.0	98.5 ± 2.0
	LG	99 ± 1.3	99 ± 1.0	97.8 ± 2.2	97.5 ± 2.6	98.6 ± 1.1	98.6 ± 1.5	99 ± 0.7
	KG	99.5 ± 0.5	99.2 ± 1.1	99.2 ± 0.4	98.8 ± 0.9	99.3 ± 0.5	98.7 ± 1.0	99.1 ± 0.8
	LMG	99.3 ± 0.7	99.2 ± 1.1	98.7 ± 1.7	98.2 ± 2.1	98.7 ± 1.9	99.2 ± 0.7	99.3 ± 1.0
	KMG	99.2 ± 1.0	99.1 ± 0.8	98.3 ± 1.6	99.1 ± 0.8	98.7 ± 0.8	98.8 ± 0.9	99 ± 0.5
	LKMG	99.5 ± 0.5	99.6 ± 0.5	99.2 ± 0.8	99.5 ± 0.7	99.2 ± 0.8	99.6 ± 0.7	99.6 ± 0.7
Etiso (V%)	CG	1.4 ± 0.2	1.4 ± 0.1	1.4 ± 0.2	1.4 ± 0.1	1.4 ± 0.4	1.2 ± 0.2	0.7 ± 0.2
	MG	0.8 ± 0.1	1.3 ± 0.4	1.4 ± 0.6	1.6 ± 0.2	1.7 ± 0.2	1.6 ± 0.2	0.9 ± 0.4
	LG	1.4 ± 0.2	1.4 ± 0.3	1.3 ± 0.1	1.3 ± 0.2	1.2 ± 0.1	1.1 ± 0.1	1.0 ± 0.2
	KG	1.2 ± 0.1	1.2 ± 1.1	1.3 ± 0.1	1.3 ± 0.1	1.3 ± 0.2	1.2 ± 0.2	1.0 ± 0.2
	LMG	1.4 ± 0.1	1.3 ± 0.1	1.3 ± 0.07	1.4 ± 0.1	1.3 ± 0.08	1.3 ± 0.1	1.3 ± 0.1
	KMG	1.4 ± 0.2	1.4 ± 0.1	1.2 ± 0.1	1.3 ± 0.1	1.3 ± 0.1	1.3 ± 1.09	1.2 ± 0.1
	LKMG	1.4 ± 0.2	1.2 ± 0.1	1.2 ± 0.1	1.2 ± 0.1	1.2 ± 0.2	1.1 ± 0.1	0.9 ± 0.2
ETCO_2_	CG	33.1 ± 8.6	34.6 ± 8.9	34.6 ± 6.6	35 ± 6.6	35.8 ± 6.8	35.3 ± 6.8	34.6 ± 7.1
	MG	33.9 ± 3.8	37.4 ± 5.7	38.4 ± 6.2	38.4 ± 5.6	38.5 ± 5.4	39.8 ± 5.8	40.3 ± 5.7
	LG	26 ± 7.7	28.8 ± 7.7	27.8 ± 8.7	26.9 ± 9.7	27.1 ± 10	29.5 ± 10.3	29.1 ± 11.7
	KG	34.5 ± 6.2	35.6 ± 8.6	33.6 ± 9.9	30.6 ± 9.7	31.8 ± 7.9	32.8 ± 7.9	31.3 ± 8.2
	LMG	27.5 ± 4.7	30.5 ± 8.8	31 ± 9.3	29.7 ± 8.9	30.5 ± 8.6	30.9 ± 9.1	30.6 ± 9.8
	KMG	27.7 ± 10.2	29.5 ± 8.3	32.8 ± 9.9	34.6 ± 11.6	34.1 ± 9.5	34.4 ± 9.2	34.2 ± 9.8
	LKMG	32.5 ± 9.3	34.2 ± 8.9	37 ± 10.1	37.8 ± 10.2	36.3 ± 9.3	36.4 ± 10.4	36.3 ± 8.8
T (°C)	CG	37.6 ± 0.8	37.6 ± 0.5	36.9 ± 0.6	36.6 ± 0.8	36.5 ± 0.8	37.0 ± 0.6	37.0 ± 0.7
	MG	37.8 ± 0.9	38.0 ± 0.6	37.9 ± 0.5	37.8 ± 0.5	37.7 ± 0.5	37.7 ± 0.5	37.7 ± 0.6
	LG	37.3 ± 0.4	37.1 ± 0.4	36.9 ± 0.4	36.7 ± 0.4	36.4 ± 0.5	36.2 ± 0.4	36.7 ± 0.5
	KG	37.7 ± 0.4	37.4 ± 0.4	37.2 ± 0.4	36.8 ± 0.4	36.3 ± 0.6	36.3 ± 0.4	36.0 ± 0.3
	LMG	37.0 ± 0.8	36.8 ± 0.7	36.6 ± 0.7	36.4 ± 0.8	36.2 ± 0.8	36.1 ± 0.9	35.8 ± 0.9
	KMG	37.2 ± 0.5	37.0 ± 0.5	36.9 ± 0.6	36.5 ± 0.6	36.5 ± 0.6	36.4 ± 0.4	35.9 ± 0.4
	LKMG	37.7 ± 0.5	37.4 ± 0.4	37.2 ± 0.4	36.9 ± 0.4	36.6 ± 0.5	36.2 ± 0.4	35.8 ± 0.5

*CG* control group; *MG* maropitant group; *LG* lidocaine group; *LMG* lidocaine and maropitant group; *KG* ketamine group; *KMG* ketamine and maropitant group; *LKMG* lidocaine, ketamine, and maropitant group. *T* esophageal temperature; *RR* respiratory rate; *HR* heart rate; *SpO*_*2*_ oxygen saturation; *Etiso* expired isoflurane concentration; *EtCO*_*2*_ partial pressure of carbon dioxide at the end of expiration; *SBP* systolic blood pressure

*Significant difference relative to CG

^#^Significant difference between MG and treatment groups

•Significant difference between LG and treatment groups. *bmp* beats per minute; *mpm*, movement per minute. M1, preoperatively; M2, after incision of the linea alba; M3, after right pedicle clamping; M4, after left pedicle clamping; M5, after ligation of the uterus body; M6, suture of the abdominal muscles; and M7, at the end of the surgery

SBP, SpO_2_, EtCO_2_, Etiso, and T showed no significant difference between the groups. These variables presented the following minimum and maximum values: SBP, 55–133 mmHg; SpO_2_, 97–100%; EtCO_2_, 35–45 mmHg; Etiso, 1–2 V%, and T, 35–38.8 °C.

**Fig. 1:**
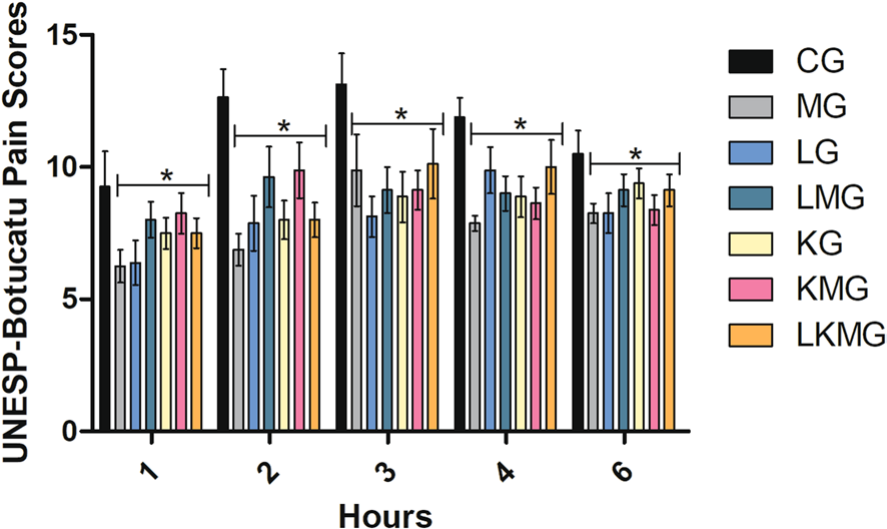
Mean ± standard error of pain scores in the UNESP-Botucatu multidimensional cat pain assessment scale after ovariohysterectomy. *CG* control group; *MG* maropitant group; *LG* lidocaine group; *LMG* lidocaine and maropitant group; *KG* ketamine group; *KMG* ketamine and maropitant group; *LKMG* lidocaine, ketamine, and maropitant group. *Significant (P < 0.05) compared to the CG. 1: 1 h; 2: 2 h; 3: 3 h; 4: 4 h; 5: 5 h, and 6: 6 h after extubation

**Fig. 2:**
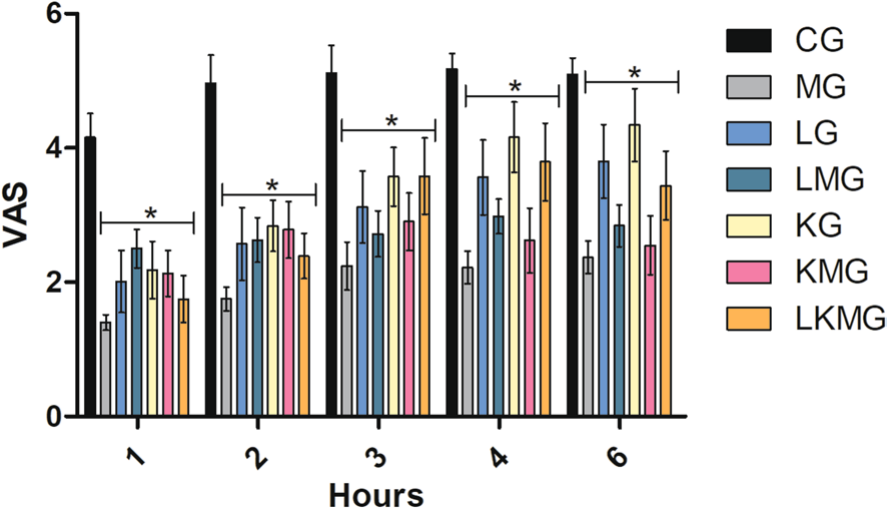
Mean ± standard error of the visual analog scale scores. *CG* control group; *MG* maropitant group; *LG* lidocaine group; *LMG* lidocaine and maropitant group; *KG* ketamine group; *KMG* ketamine and maropitant group; *LKMG* lidocaine, ketamine, and maropitant group; *VAS* visual analogue scale. *Significant (P < 0.05) compared with CG. 1: 1 h; 2: 2 h; 3: 3 h; 4: 4 h; 5: 5 h, and 6: 6 h after extubation

The pain scores were lower in the MG group than in the CG group at all assessment time points for both scales used. The pain score of MG group did not differ from the other groups that received lidocaine or ketamine alone and their combinations, at any time point. Pain scores in the other groups differed statistically from that of the CG group (P = 0.01) (Figs. 1 and 2). Animals in the CG group (28 rescue) needed more postoperative analgesic rescue than those in the other groups (rescues: MG, 3; LG, 9; KG, 11; LMG, 14; KMG, 17; LKMG, 15) (P = 0.01). The animals in the LMG (14 rescues), KMG (17 rescues), LKMG (15 rescues) groups required more postoperative analgesic rescues than the MG (3 rescues).

## Discussion

The administered doses of maropitant (IV bolus and continuous IV infusion) were obtained from the literature [17]. The bolus dose of lidocaine and the infusion dose were extrapolated from that commonly used in dogs [7, 20–22], although higher doses can be used [7, 20–22]. The lower dose was used as some studies have questioned the use of lidocaine in cats [10, 23]. Also, the doses of ketamine were reduced compared to those used in other studies on cats [24] and dogs [7, 20] as high doses of ketamine can increase SBP and HR in domestic cats [24].

Cats belonging to MG, LG, and KG had lower HR and RR than those in the CG group, especially at the time of ovarian pedicle traction. Mesovary traction and ligation generate noxious stimuli, which can increase the HR and RR due to the autonomic nervous system response [25, 26]. In this study, a reduced intraoperative autonomic response at the time of greatest nociceptive stimulus indicated decreased sympathetic response to the surgical stimulus due to the antinociceptive effect of the drugs used.

Cats that received lidocaine alone or in combination with other drugs were stable in HR, SBP, and RR. The plasma concentration of lidocaine was probably < 5 µg/mL, and therefore at a level not expected to influence HR or SBP [10]. Pypendop and Ilkiw [10] observed a marked depression of the cardiovascular system in domestic cats, and therefore, did not recommend the use of IV lidocaine for multimodal anesthesia in this species [10]. The observed difference may be due to the IV bolus and continuous IV infusion doses used in this study. The doses used in our study were lower for IV bolus and three times lower for continuous IV infusion when compared to doses used by Pypendop and Ilkiw [10]. We reduced the doses, as lower doses can lead to a lower incidence of cardiorespiratory changes.

In the present study, HR and SBP remained unaltered despite the use of ketamine as also observed by Gutierrez-Blanco et al. [20], but different from the findings by Pascoe et al. [24]. The latter study reported an increase in HR and SBP during continuous IV infusion with ketamine in domestic cats. It should be noted that the doses used by Pascoe et al. [24] (IV bolus, 2 and 6 mg/kg; continuous IV infusion, 46 and 115 µg/kg/min) were higher than those used in our study. Boscan et al. [27] observed an increase in HR and SBP in dogs during continuous IV infusion of ketamine. Continuous IV infusion of low doses of ketamine, mainly for analgesia, does not induce critical effects on HR and SBP, or induce respiratory depression in cats and dogs [23].

Continuous IV infusions of maropitant, ketamine, lidocaine, and their combinations did not significantly change SpO_2_, T, and ETCO_2_. These results are in congruence with findings reported previously [20, 21] for continuous lidocaine infusion in dogs, and continuous maropitant or ketamine infusion in cats or dogs [17, 24, 28–30].

All treatment groups showed an antinociceptive effect when compared with CG. It has been shown that isolated or combined administration of drugs reduce pain, indicated by the low numbers of analgesic rescues and lower scores in the pain assessment scales. Lidocaine and ketamine are indicated for the management of acute pain in dogs resulting from surgical procedures [11]. IV administration of these drugs in cats, however, is still a recent practice [11, 31]. Since maropitant is an NK1 receptor antagonist, it has demonstrated an antinociceptive effect in cats [17] and an anti-inflammatory effect in rats [32].

When analgesic drugs are not used, nociceptive signaling generated during surgical procedures induce central sensitization as well as neuroplasticity in the dorsal horn of the spinal cord [1, 33]. Central sensitization results from increased and persistent excitability of the neurons, especially the C-fibers, and comprises multiple mechanisms involving excitatory neurotransmitters, their receptors, and ion channels. Among them are the neurotransmitters substance P and glutamate, and their receptors NK1 and NMDA, respectively [34]. The use of drugs for multimodal analgesia prevents the activation of these receptors, consequently avoiding central sensitization, promoting pain relief during the postoperative period, and preventing the development of chronic pain [35]. IV lidocaine interacts with sodium channels at peripheral and central nerve endings [36]; ketamine blocks the NMDA receptors [1]; and maropitant blocks the NK1 receptors [13]. Thus, these mechanisms justify the antinociceptive effect observed in this study.

Continuous IV infusion of maropitant, lidocaine, or ketamine alone promoted an antinociceptive effect. In dogs, continuous infusions of ketamine and lidocaine alone or in combination or with other drugs induce antinociceptive effects and decrease analgesic requirements [7, 37]. The groups that received combinations did not significantly differ from the group that received only maropitant. We believe that maropitant may have interfered with the mechanism of action of ketamine and lidocaine [38, 39] and that is why the combinations required a greater number of rescues. Further studies are needed to determine whether maropitant can alter or compete with other drugs in ion channels and the NMDA receptor.

Our study shows that ketamine produced an isolated antinociceptive effect, probably because the IV bolus administration, followed by continuous IV infusion throughout the surgical procedure, maintained adequate plasma concentrations for sustained analgesia. A study of ketamine (0.5 mg/kg) as the pre-anesthetic medication in cats showed that ketamine alone does not produce satisfactory analgesia postoperatively [40]. We believe that the use of a single bolus dose may be insufficient to maintain adequate plasma concentration for sustained analgesia; thus, continuous IV infusion can resolve this issue. In dogs, very low plasma levels of ketamine are ineffective in promoting antinociceptive effects [37, 41].

Although Kinobe and Miyake [42] reported that maropitant reduces the minimum alveolar concentration of inhaled anesthetics, but without analgesic and anti-inflammatory activity, their systematic review and meta-analysis highlights the limited number of studies, requiring more targeted research to prove whether maropitant has such effects. IV infusions of drugs during elective surgical procedures aim at an antinociceptive effect, as there is no previous pain [2]. Thus, both lidocaine, ketamine and maropitant in this study promoted an antinociceptive effect, because they acted by blocking receptors in the pain pathway, preventing central sensitization. The reduction in pain scores in the postoperative period of the treated animals was due to the antinociceptive action of each drug used.

Maropitant produced a postoperative antinociceptive effect in cats undergoing OHE. This effect was also observed with the administration of lidocaine and ketamine alone, which is consistent with the findings of Corrêa et al. [17].

## Conclusion

Continuous IV infusion of maropitant, lidocaine and ketamine alone in domestic cats generated an analgesic effect in the postoperative period after OHE. Combination of these drugs, however, did not significantly increase the analgesic effects. The study provides insight into the use of these drugs for multimodal analgesia in cats, mainly by demonstrating that maropitant, lidocaine and ketamine did not generate adverse effects or other types of alterations indicative of intoxication.

## Data Availability

The datasets used and/or analysed during the current study are available from the corresponding author on reasonable request.
